# Novel Cleaning-in-Place Strategies for Pharmaceutical Hot Melt Extrusion

**DOI:** 10.3390/pharmaceutics12060588

**Published:** 2020-06-24

**Authors:** Martin Spoerk, Ioannis Koutsamanis, Josip Matić, Simone Eder, Carolina Patricia Alva Zúñiga, Johannes Poms, Jesús Alberto Afonso Urich, Raymar Andreína Lara García, Klaus Nickisch, Karin Eggenreich, Andreas Berghaus, Kathrin Reusch, Yorick Relle, Johannes Khinast, Amrit Paudel

**Affiliations:** 1 Research Center Pharmaceutical Engineering GmbH, Inffeldgasse 13, 8010 Graz, Austria; ioannis.koutsamanis@rcpe.at (I.K.); josip.matic@rcpe.at (J.M.); simone.eder@rcpe.at (S.E.); carolina.alva@rcpe.at (C.P.A.Z.); johannes.poms@rcpe.at (J.P.); jesus.afonso@rcpe.at (J.A.A.U.); lara.raymar@rcpe.at (R.A.L.G.); khinast@tugraz.at (J.K.); 2 Evestra Inc., 6410 Tri County Parkway, Schertz, TX 78154, USA; knickisch@evestra.com (K.N.); keggenreich@evestra.com (K.E.); 3 ColVisTec AG, Max-Planck-Straße 3, 12489 Berlin, Germany; a.berghaus@colvistec.de; 4 Leistritz Extrusionstechnik GmbH, Markgrafenstraße 38, 90459 Nuremberg, Germany; knickel@leistritz.com (K.R.); yrelle@leistritz.com (Y.R.); 5 Institute of Process and Particle Engineering, Graz University of Technology, Inffeldgasse 13, 8010 Graz, Austria

**Keywords:** cleaning verification, cleaning-in-place, hot melt extrusion, API contamination, UV–Vis spectroscopy, process analytical technology, swab and rinse tests, estradiol, estriol, ibuprofen

## Abstract

To avoid any type of cross-contamination, residue-free production equipment is of utmost importance in the pharmaceutical industry. The equipment cleaning for continuous processes such as hot melt extrusion (HME), which has recently gained popularity in pharmaceutical applications, necessitates extensive manual labour and costs. The present work tackles the HME cleaning issue by investigating two cleaning strategies following the extrusion of polymeric formulations of a hormonal drug and for a sustained release formulation of a poorly soluble drug. First, an in-line quantification by means of UV–Vis spectroscopy was successfully implemented to assess very low active pharmaceutical ingredient (API) concentrations in the extrudates during a cleaning procedure for the first time. Secondly, a novel *in-situ* solvent-based cleaning approach was developed and its usability was evaluated and compared to a polymer-based cleaning sequence. Comparing the in-line data to typical swab and rinse tests of the process equipment indicated that inaccessible parts of the equipment were still contaminated after the polymer-based cleaning procedure, although no API was detected in the extrudate. Nevertheless, the novel solvent-based cleaning approach proved to be suitable for removing API residue from the majority of problematic equipment parts and can potentially enable a full API cleaning-in-place of a pharmaceutical extruder for the first time.

## 1. Introduction

In the pharmaceutical industry, proper cleaning of production equipment is of paramount importance in order to prevent cross-contamination of drug products [1,2] and avoid equipment malfunctions [3]. Two approaches exist to control the cleaning processes: cleaning validation and cleaning verification (CV) [3]. Cleaning validation is a documented process that assesses the effectiveness and consistency of pharmaceutical equipment cleaning for a large number of compounds manufactured at the respective site [4]. It is an extensive multi-functional programme, which considers the entire manufacturing process. In contrast, CV is performed upon the completion of a process for each piece of manufacturing equipment to determine if the drug’s presence is below a set acceptance level. Typically, CV confirms the effectiveness of the cleaning procedure and is applied when no established equipment cleaning procedure exists [3], e.g., during manufacturing products for phases I and II of clinical trials.

Every CV procedure consists of two steps: sampling and detection. Sampling is made via swabbing and/or rinsing [3]. During swabbing, an inert material is rubbed into a part of the equipment’s surface [5]. The selected surfaces are assumed to represent the entire line, which can be challenging to justify for complex processes. Rinsing is applied at sites that are difficult to access, and it is assumed that all the residue is rinsed [1]. Both methods have certain drawbacks. First, the swabbing procedure can be very user-dependent and, thus, needs to be validated in advance [5]. Secondly, the active pharmaceutical ingredients (API(s)) must be sufficiently soluble in the solvent applied in both swab [6] and rinse [1] sampling. Thirdly, both are indirect methods, which may result in incomplete residue recovery. Finally, sample collection and analysis are time-consuming. For example, swabbing and a high-performance liquid chromatography (HPLC) analysis, which is most commonly used for detection in cleaning studies [7], can take several hours. Hence, the verification of the cleaning procedure following each cleaning step is cumbersome, while the FDA requires continued process verification [8]. Without continuous process verification, the risk of cross-contamination may be considerably increased [9].

This demonstrates that the pharmaceutical industry benefits from rapid in-line and at-line strategies for continuous CV. Several analytical techniques have been reported in the literature, including total organic carbon (TOC) [10], ion mobility spectroscopy [11,12,13,14], mass spectrometry [15,16] and more recently a light induced fluorescence sensor for at-line CV [17]. Furthermore, near infrared (NIR) chemical imaging was shown to be suitable for CV of various surface materials [18]. These methods have advantages over the classical rinse and swab/HPLC approach, since they require considerably less time and/or can potentially eliminate the prerequisite of swabbing/rinsing. This would be particularly beneficial in complex, continuous pharmaceutical processes.

Hot melt extrusion (HME) is a continuous pharmaceutical process that has recently attracted considerable attention [19] for developing formulations of poorly soluble drugs [20], its applicability to an increasing range of materials [21] and its solvent-free process [22]. Yet, HME requires a time-consuming and labour-intensive conventional cleaning procedure [22], particularly after a change of formulation [3]. A typical cleaning process of the extruder comprises the following steps: (i) running the extruder without feeding any material until no material exits the die [22]; (ii) pre-cleaning the screws and barrels by extruding special purging or liquid cleaning materials [22,23]; (iii) disassembling the extruder die, screws, barrels and barrel openings [22]; (iv) mechanically removing the majority of polymer by scrubbing/brushing equipment [22,24]; (v) vacuum-cleaning the powder feeding inlets to remove any residual powder; (vi) soaking each piece of equipment in an appropriate solvent for several hours to remove any API and excipient residue [25]; (vii) performing the swab and rinse tests in critical locations on the extruder according to a verified procedure [5] and equipment cleaning cycle [3]; (viii) conducting an HPLC analysis; and (ix) reassembling all the parts of the extruder if they are clean. To reduce the CV effort, HME would greatly benefit from in-line processes that assess the cleanliness of the material and equipment. Although ultraviolet-visible (UV–Vis) spectroscopy has been shown to be highly applicable to in-line analyses in HME [26,27,28,29,30], e.g., for monitoring the API content [30], to date no scientific study has employed UV–Vis spectroscopy to detect the API in terms of cleanliness of a material and/or processing equipment. In addition, it is not certain, if an API-free material that is mainly analysed by in-line techniques such as UV–Vis spectroscopy, can also represent an API-free processing equipment [9], since contaminated material might be trapped in dead spots of the equipment [31] and contribute to cross-contamination. While methods for decreasing the cleaning effort for an extruder from other pharmaceutical production techniques [32,33], e.g., by applying washing- or cleaning-in-place strategies, or at least reducing the number of cleaning steps would be very useful, they are still scarce for continuous processes such as HME [34].

The present study aims at closing these gaps by analysing various cleaning procedures for a hot melt extruder by means of conventional swab and rinse tests and in-line UV–Vis spectroscopy. The cleanability of highly potent APIs and a typical sustained-release formulation is presented as a function of different cleaning sequences. As a result, a novel solvent-based cleaning-in-place strategy was developed, which has the potential to remove any API residue on the extrusion equipment *in-situ* without subsequent mechanical cleaning of the equipment.

## 2. Materials and Methods

### 2.1. Materials

Ethylene-vinyl acetate copolymer with a vinyl acetate content of 28% (Greenflex FL65, EVA) supplied by Ter Hell & Co. GmbH, Hamburg, Germany as pellets was ground to powder externally. Poly(ethyl acrylate-*co*-methyl methacrylate-*co*-trimethylammonioethyl methacrylate chloride) 1:2:0.2 (Eudragit RL-PO, EUD) was purchased from Evonik Nutrition & Care GmbH, Essen, Germany. HME Cleaner Plus (CleanPoly, Biogrund GmbH, Hünstetten, Germany) containing hydroxypropyl methylcellulose, methylcellulose, propylene glycol and amorphous silicon dioxide was used as a cleaning polymer specialised for HME. 17β-estradiol (E2) was purchased from Bayer Schering AG, Leverkusen, Germany and estriol (E3) was obtained from Shenzhen Nexconn Pharmaceuticals Ltd., Shenzhen, China. Ibuprofen 25 (IBU) was supplied by BASF SE, Ludwigshafen, Germany. For the swab-based sample collection, the swabbing rods 149-0264 (VWR International, LLC, Radnor, PA, USA) of polyurethane foam with a length of 131 mm were used. Unless stated otherwise, ethanol (96 vol.-%, Sigma-Aldrich, St. Louis, MO, USA), acetonitrile (HiPerSolv Chromonorm^®^, VWR International, LLC, Radnor, PA, USA) or water purified by TKA MicroPure UV (JWT GmbH, Jena, Germany) served as solvents.

### 2.2. Processing

#### 2.2.1. Polymer-API Extrusion

**Figure 1:**
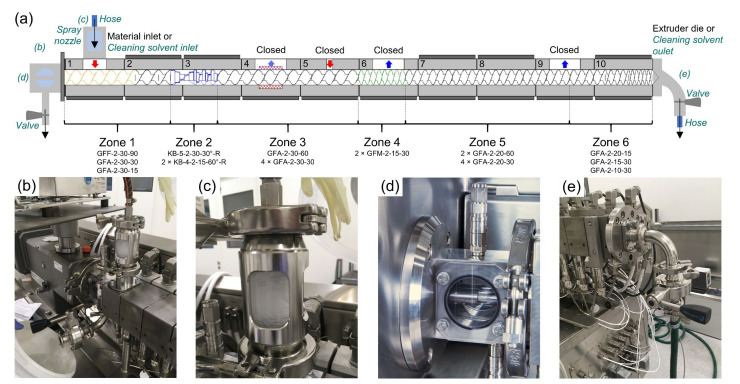
Screw configuration used (**a**) and detailed images of the optional solvent-cleaning equipment (**b**–**e**). In (**a**), the equipment for the additional solvent-cleaning step for the formulation EUD/IBU_10% is indicated in green italic letters. The standard extrusion equipment used for every formulation is labelled in black letters, with the barrels numbered from 1 to 10 and the screw zones designated as zones 1–6. The respective screw elements of each zone are given according to the nomenclature of the equipment producer (Leistritz Extrusionstechnik GmbH, Nuremberg, Germany), with GFF referring to non-self-wiping conveying elements with an enlarged free volume, GFA to intermeshing self-cleaning conveying, KB to kneading and GFM to mixing elements.

The investigated formulations, the API type and amount in the formulations (Table 1) were selected based on typical API loadings for hormone drug delivery reported in the literature [35] and clinical studies [36] and for a representative amorphous solid dispersion [37]. All physical powder blends were manually sieved and blended batchwise for 30 min at 60 Hz (Turbula, Willy A. Bachofen AG, Muttenz, Switzerland). The resulting powder mixtures were fed via the gravimetric twin-screw pharma feeder K-Tron KT20 (Coperion GmbH, Stuttgart, Germany) into the ZSE18 twin screw extruder equipped with an adapter plate (both Leistritz Extrusionstechnik GmbH, Nuremberg, Germany) and a die with a diameter of 4 mm. Since the processed formulations are binary mixtures, a screw configuration that does not introduce extensive amount of shear was selected (Figure 1a). The arrangement and choice of the kneading blocks guaranteed a soft back-conveying section with enhanced distributive mixing capabilities, allowing a sufficient local residence time for melting the formulations [38,39,40]. Consequently, any potential API agglomerate that may have formed in the hopper breaks up, which guarantees a uniform API distribution in the polymer matrix. In addition, in order to prevent any thermal degradation, two consecutive mixing elements were chosen to increase distributive mixing without introducing high viscous dissipation [22,38,40]. All open barrels for side/top feeding (barrel 4 and 5 in Figure 1a) or de-gassing (barrel 6 and 9 in Figure 1a) remained closed by closing inserts. The extrudate was pulled by the haul-off unit Primo 60E (Maag Automatik GmbH, Großostheim, Germany) through a water bath and was stored in the fridge at 5 °C until subsequent characterisations were performed. 

The detailed extrusion cleaning process sequences and the respective settings for each formulation are summarised in Table 2 and Table 3. To keep the start-up torque below the equipment limit, an extrusion start-up procedure was applied, allowing a gradual increase in the process throughput and the screw speed. As soon as the intended process settings were achieved, the actual process time started. To guarantee a stable process in terms of pressure and torque, the total process time was set to 30 min.

#### 2.2.2. Polymer-Based Cleaning

After the main API extrusion, the extruder was emptied by continuing the extrusion for 3 more minutes with a lower screw speed without feeding additional material. In the meantime, the feeder was cleaned and refilled solely with the excipient, while the barrels remained heated. As soon as pure excipient was fed, the first cleaning step of the polymer-based cleaning sequence began. The segments of the extrudate were gathered every 30–60 s for a subsequent analysis of the API content of the extrudate (Section 2.6). In addition, for EVA/E2_1% and EVA/E3_5%, the decreasing API content during this cleaning step was monitored in-line via UV–Vis spectroscopy (Section 2.3). Since IBU acted as a plasticiser [41,42,43], the temperature of selected barrels was increased during the pure excipient extrusion to compensate for an increase in the viscosity.

During this first cleaning step with the pure excipient, the throughput and the screw speed were adjusted to ensure different extruder filling degrees and residence time (RT) distributions. The first step mirrored the previous API-extrusion settings (1.9 kg·h^−1^, 300 rpm), while the second one resulted in a high degree of filling and a long RT (3.0 kg·h^−1^, 150 rpm). The last step led to a low degree of filling and a short RT (1.5 kg·h^−1^, 400 rpm). The idea behind pre-cleaning the extruder only with excipient was to clean the API in possible dead zones using a compound whose specific volume is similar to that of the base excipient-API mixture by establishing comparable RT distributions and screw filling degrees. After the pure excipient extrusion, the barrels were heated to 160 °C to counteract the high viscosity of the cleaning polymer. The feeder was removed and the cleaning polymer was fed manually by flood feeding to result in high screw fillings, as automatic feeding resulted in substantial bridge formations in the material inlet. Changes in the screw speed and the feeder throughput were introduced in a similar way as in the pure excipient run in order to additionally clean the screw and the barrel by altering the screw filling degree. After feeding the cleaning polymer, the screws were emptied by continuing the extrusion without adding any more material until no extruder torque reduction was observed. For all three formulations, the polymer-based cleaning procedure was finished after the cleaning polymer extrusion by removing the die section and the screws. During this step, special care was taken not to remove any polymer/powder from the screw elements to avoid influencing the subsequent swab and rinse analyses. The screw elements and the extruder barrels were cooled down to room temperature, upon which the rinse and swab tests were performed (Section 2.4).

#### 2.2.3. Solvent-Based Cleaning

For EUD/IBU_10%, an additional solvent-based cleaning sequence was performed directly after the polymer-based cleaning sequence. This step should realise a polymer- and API-free extruder barrel and screw without removing the screw and mechanically cleaning the extruder components. In order to flood the extruder barrel with the desired solvent, a special cleaning equipment designed by Leistritz Extrusionstechnik GmbH, Nuremberg, Germany was installed to allow cleaning-in-place. Instead of the material hopper in barrel 1 (Figure 1a), a cleaning solvent inlet was introduced consisting of a hose connected to the solvent source, a rotating spray nozzle (Schlick model 300 size 0, Düsen-Schlick GmbH, Untersiemau, Germany) that distributed the solvent around the opening of barrel 1, a capacity of approximately 500 mL and an observation window (Figure 1c). The extruder’s die was replaced by the cleaning solvent outlet that was connected to a hose running into the solvent container. The solvent outflow amount was controlled via an exit-valve (Figure 1e). Additionally, a washable, sealed lantern around the screw shafts was installed to gather the overflowing amount of solvent from the process section through the shaft sealings. This amount was controlled using an observation window (Figure 1d) as well. If necessary, the overflowing solvent could be removed by opening the side outlet valve (Figure 1b), and the whole lantern could be washed by flushing all connections.

First, deionised water was flushed through the extruder at a constant flow rate of 255 ± 1 mL·s^−1^ by connecting the general water supply with the solvent inlet in barrel 1 of the extruder. Special care needs to be taken that the water flow rate is sufficiently high to guarantee a continuous rotation of the spray nozzle. The extruder was operated at high screw speeds (Table 3) and at room temperature, while the exit-valve and the side outlet valve were open. This water-based step was performed under high screw speeds and unheated barrels to remove most of the remaining water-soluble cleaning polymer [44], and the majority of other residue by applying high shear forces as well as to prevent any backflow from the screws to the spray nozzle. Secondly, the hose of the solvent inlet was removed, the exit- and side outlet valves were closed, the barrels were heated up to 60 °C to improve the API solubility, and the extruder was manually filled with a 50/50 wt.-% solution of deionised water and ethanol. The extruder was soaked in this solution at a constant screw rotation in order to remove the majority of ibuprofen, which is highly soluble in the present solvent [45]. Slower screw rotations were employed to keep the pressure on the closed exit-valve low and to prevent the temperature to exceed above the boiling point of ethanol. Next, the exit-valve was opened and the solvent was gathered for a subsequent HPLC analysis. Thirdly, the flushing sequence with water was repeated for another 10 min. To remove the remaining water, the empty extruder barrels were heated to 120 °C and operated with all valves open. After cooling down the barrels and removing the solvent-cleaning equipment and the screws, the swab and rinse tests were performed.

### 2.3. UV–Vis Inline API Determination

For the in-line API analysis of EVA/E2_1% and EVA/E3_5%, the UV–Vis spectrophotometer InspectroX equipped with two transmission polymer melt probes (both ColVisTec AG, Berlin, Germany) was attached to the extruder die in axial alignment, as reported elsewhere [26,29]. The measurements were performed in transmission between 250 and 400 nm with a sampling rate of 0.167 Hz. To enhance the UV sensitivity by suppressing the peaks of xenon flashes in the visible light range and preventing the sensor from oversaturation, the spectrophotometer was modified via the UV UG-5 bandpass filter (Edmund Optics GmbH, Mainz, Germany). For each measurement, 20 individual spectra were averaged. For each spectrum, 7 xenon lamp flashes were used to illuminate the sample. The inline calibration was performed during the extrusion of pure EVA at the initial temperatures, throughput and screw speed, i.e., T_B5-10_ = 120 °C, ṁ = 1.9 kg·h^−1^ and *n* = 300 rpm, respectively. The inline measurements were performed at the same process conditions and temperatures. For both EVA-based formulations (EVA/E2_1%, EVA/E3_5%), offline calibration samples of known concentrations were obtained using the vacuum compression moulding (VCM) Spectroscopy Chamber (MeltPrep GmbH, Graz, Austria) and attached probes using VCM. A split chemometric model was created including two concentration ranges. In the regime of low API concentrations (<0.05 wt.-%), in which the whole absorption spectrum was within the dynamic range of the detector, a partial-least-squares model was employed to predict the API concentration using all wavelengths. In the regime of higher concentrations, in which the absorption peak was higher than the dynamic range of the detector, a procedure similar to Ref. [27] was performed on a subset of wavelengths. To evaluate the latter regime, an integration of the shoulder of the absorption peak between 300 and 370 nm was proportional to the concentration and, thus, used for the prediction. Based on the low-concentration chemometric model, the limits of detection (LOD) and quantification (LOQ) for E2 were determined by the signal to noise ratio method of blank spectra as 0.005 wt.-% and 0.015 wt.-%, respectively.

To confirm the solid state of the hormones in EVA at the processing temperature of 120 °C, thin cryo-cuts of the extrudates (manual microtome MT.5503, Euromex, Arnheim, The Netherlands) were investigated by means of polarised optical microscopy (Olympus BX51M, Olympus K.K., Tokyo, Japan) with a temperature-controlled stage (THMS 600/720, LINKAM Scientific Instruments, Tadworth, UK) as described in [35,46].

### 2.4. Rinse Tests

After the last cleaning step, the screw elements were separated, cooled down and grouped according to their zones (Figure 1a). The grouped screw elements were transferred into glass beakers of suitable sizes. Sufficient rinse solvent was added to fully immerse the screw elements and to ensure constant ratios of the screw elements’ total surface area (calculated using the software SolidWorks 3D CAD, Dassault Systèmes, Vélizy-Villacoublay, France) to the solvent’s volume. The rinse solvent was chosen considering a high API solubility and a high solubility/swelling capacity of the cleaning polymer in the solvent. Consequently, ethanol was used in the rinse tests for EVA/E2_1% and EVA/E3_5% [35]. A 50/50 wt.-% solution of deionised water and ethanol was used for EUD/IBU_10% [45]. The filled glass beakers were sealed with parafilm and stored under ambient conditions for at least 18 h without external agitation. Subsequently, the screw elements were removed and the API in the solvent was quantified by means of HPLC (Section 2.6).

To verify that all of the API was extracted by the rinse solvent and to mimic the worst possible cleaning scenario, recovery tests were performed for extruded formulations containing cleaning polymer and the respective amount of API used in the main formulations (CleanPoly/E2_1%, CleanPoly/E3_5% and CleanPoly/IBU_10%). A suitable amount of solvent (ethanol for CleanPoly/E2_1% and CleanPoly/E3_5%; 50/50 wt.-% solution of deionised water and ethanol for CleanPoly/IBU_10%) which was determined based on the constant ratio between the surface area and the solvent’s volume calculated for the rinse tests, was added to 220 ± 20 mg of extrudate. After 10, 14 and 18 h of rinsing the extrudates without external agitation, samples were withdrawn, diluted in a 50/50 vol.-% solution of purified water and acetonitrile and analysed by means of HPLC (*n* = 3 for each time point).

### 2.5. Swab Tests

**Figure 2:**
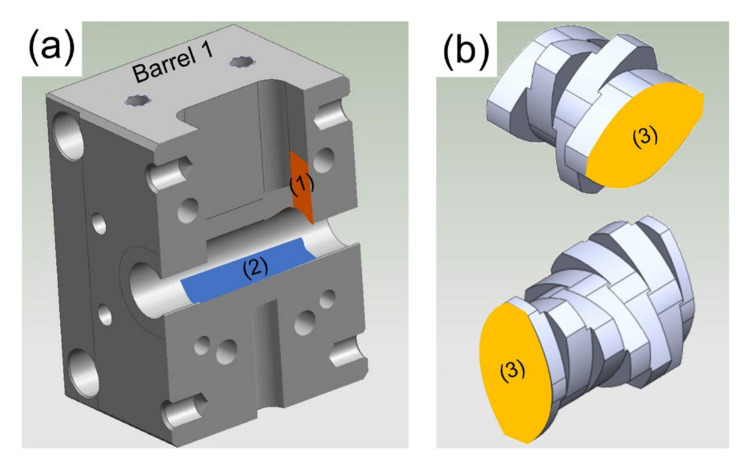
Swabbing locations on one representative barrel (**a**) and screw elements (**b**). The marked areas and their abbreviations refer to the swabbing locations of the barrel entrance (1), barrel channel (2) and in between adjacent screw elements of one screw (3).

To investigate the areas in the extruder that may not have been rinsed properly, the following barrel locations with high contamination potential were swabbed directly after the extrusion process (Figure 2a): the lower part of the surface of the entrances of barrels 1, 6 and 9 (Figure 2a location (1)) and the depression surface of the channels of barrels 1, 5 and 10 (Figure 2a location (2), Figure 1a). In the case of barrel channels, one swab was taken from the channel of each screw. As for the barrel entrances, swabs were taken from the entrance surfaces perpendicular to the extrusion direction that face each other (Figure 2a location (1)). All locations were swabbed without dismounting the barrels. Hence, prior to swabbing, the covers of barrels 5, 6 and 9 were detached. Additionally, selected surfaces between adjacent screw elements of one screw (Figure 2b location (3)) were swabbed prior to rinsing them, i.e., the surface between the GFF-2-30-90 (GFF) and GFA-2-30-30 (GFA) elements in screw zone 1 and the surface between the KB-5-2-30-30°-R (KB30°) and KB-4-2-15-60°-R (KB60°) elements in screw zone 2 (Figure 1a).

The general procedure of taking swabs was based on ref. [5]. Based on pre-defined swabbing procedures/patterns, the tests were performed by the same person in the following manner: Prior to sampling, a new swabbing rod was pre-moistened in ethanol. After removing any excess solvent by squeezing the sides of the swabbing rod, the respective surface was sampled in similar surface areas (~25 cm^2^) by applying a constant gentle downward pressure and following a consistent sampling pattern (i.e., swabbing one side of the swab with overlapping patterns, flipping the swab and repeating the same pattern in perpendicular direction using the other side of the same swab). The swab’s handle was then cut off and placed into a glass vial. After evaporating the remaining solvent from the swab, the glass vial was closed with parafilm and stored under ambient conditions for maximum 5 days.

Depending on the API to be analysed, two extraction procedures were used. To analyse E2 and E3, the swabs were transferred into 15 mL crimp top vials. Subsequently, 5 mL of ethanol were added, and the vials were placed in an incubator shaker operating at 200 rpm for 1 h at room temperature. After the solid particles settled, the supernatant was withdrawn, centrifuged for 10 min at 15,000 rpm and transferred into HPLC glass vials. To analyse ibuprofen, the swabs were immersed in 10 mL of a 50/50 vol.-% solution of purified water (HPLC grade) and acetonitrile in crimp top vials. After immersing the samples in an ultrasonic bath (Elmasonic S 30/H, Elma Schmidbauer GmbH, Singen, Germany) for 20 min, an aliquot of 3 mL was transferred into a 5 mL volumetric flask filled up with a 50/50 vol.-% solution of purified water and acetonitrile. Next, the solutions were filtered into HPLC vials using the 0.22 µm Yeti syringe filters (Merz Brothers GmbH, Ansfelden, Austria). To exclude any material originating from the swabs, a blank swab was treated the same way as the samples and was subjected to the HPLC analysis. In order to represent the worst state of API contamination, only the highest API concentrations detected per location are considered.

### 2.6. HPLC Analysis

All API concentrations in the swabs and rinse solutions were determined using reversed-phase HPLC (Acquity UPLC System, Waters Corporation, Milford, MA, USA) and the software Empower^®^ (Waters Corporation, Milford, MA, USA). The detailed methods for each API are summarised in Table 4. The LOQ and LOD of all APIs were determined by regression analysis.

## 3. Results

### 3.1. Inline Monitoring of the API Content

**Figure 3:**
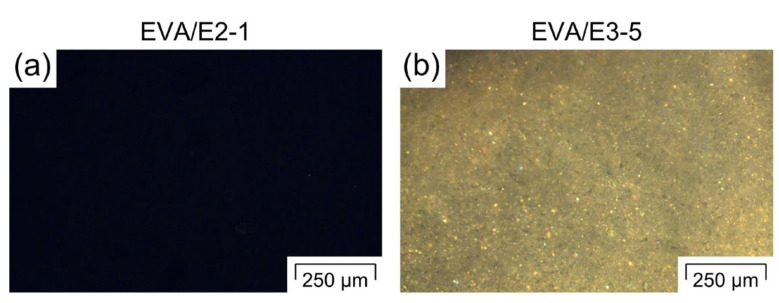
Polarised optical microscopy images of thin microtome cuts after melting EVA around 120 °C for the extrudates of EVA/E2_1% (**a**) and EVA/E3_5% (**b**).

**Figure 4:**
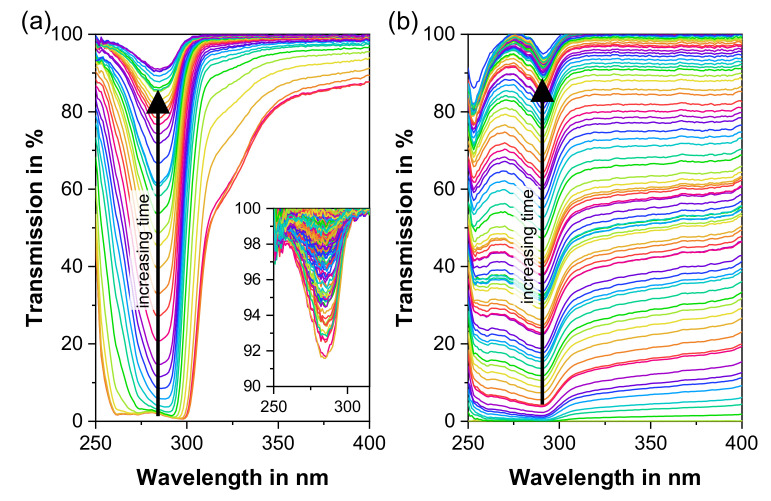
In-line ultraviolet-visible (UV–Vis) spectra of the respective peaks of E2 (**a**) and E3 (**b**) as a function of extrusion time of pure EVA for the first 10 min in the first cleaning sequence. In (**a**), the zoom represents the spectra of the time span between 4 and 10 min, whereas the main diagram in (**a**) is a timespan of the first 4 min.

The results of the in-line monitoring of the E2 and E3 concentration via UV–Vis spectroscopy during the initial step of the polymer-based cleaning sequence (Table 2, pure EVA extrusion) are presented below. For the purpose of the determination of the API dissolved/dispersed in the polymer via UV–Vis spectroscopy, the API’s solubility in the polymer plays a major role. Up to 1 wt.-% of E2 was fully soluble in EVA with VA contents of ≥28 wt.-% [35]. When exposed to shear, even higher E2 loadings were dissolved [35]. As a result, for EVA/E2_1%, no crystalline API were seen under polarised light after melting EVA, as evidenced by the lack of birefringence (Figure 3a). Macroscopically, the extrudate appears transparent. Therefore, a broad saturated peak between 250 and 350 nm with a minimum around 284 nm that relates to E2 [47] is observed in the UV–Vis spectrum (Figure 4a) in the beginning of the polymer-based cleaning sequence. With increasing extrusion time of pure EVA and increasing cleaning time, the intensity of the E2 peak steadily decreases (Figure 4a). This represents a reduction in the E2 concentration in the extrudate with time during the first step of the polymer-based cleaning sequence. Within the first 4 min of pure excipient extrusion, the peak intensity decreases drastically: from the initial API load (1 wt.-%) down to concentrations around the detection limit (0.005 wt.-%). Between 4 and 10 min of pure excipient extrusion (zoom in Figure 4a), very low quantities, i.e., <0.005 wt.-% of E2, can still be detected but not quantified via UV–Vis. However, after 6 min of extrusion, all peaks reveal a signal below the detection limit. Similarly, no distinct E2 concentration peaks characteristic for the extrusion of E2 residue are observed during the remaining cleaning process (data not shown in Figure 4a), which suggests a clean extrudate.

Since the equilibrium solubility of E3 in EVA is approximately four times lower than that of E2 at room temperature (data not shown, experiments based on ref. [48]), and since the API content is five times higher for EVA/E3_5% than for EVA/E2_1%, most of E3 is present in EVA as crystals, even at the processing temperature of 120 °C. This is confirmed by the crystalline API structures under polarised light (Figure 3b) and the white colour of the extrudates. Since the transmitted UV–Vis radiation is blocked by the non-solubilised API crystals, there is no clear absorption peak but rather a scatter at around 0% transmission in the UV–Vis signal for EVA/E3_5% in the beginning of the polymer-based cleaning sequence (Figure 4b). Oversaturated Kollidon/piroxicam formulations investigated via UV–Vis had similar behaviour [29]. As the API content decreases with increasing cleaning time, the amount of transmitted light rises steadily until most of E3 is removed. Below a certain E3 concentration, sufficient UV–Vis radiation can be transmitted to produce a peak at ~290 nm, which generally refers to the absorption band of E3. In the present case, however, its intensity is below the LOD of the method due to the presence of E3 in the crystalline form (Figure 3b). Hence, this peak cannot be used as a basis for a quantitative analysis of the API content.

**Figure 5:**
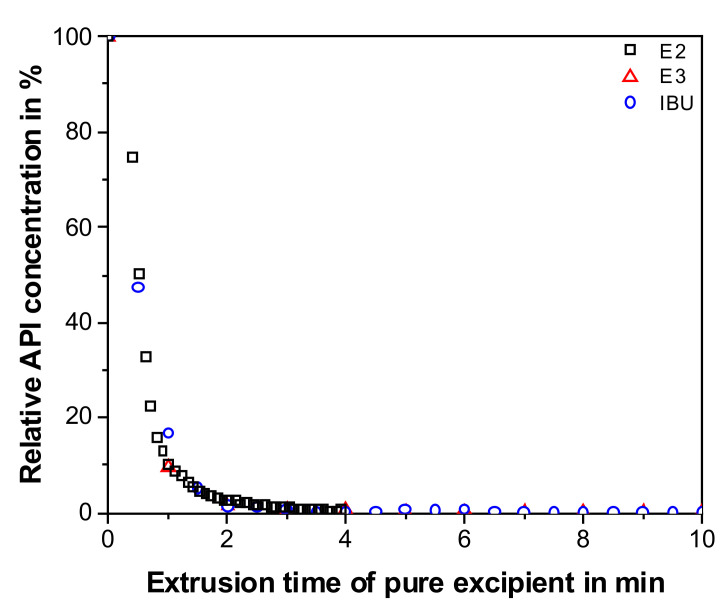
Relative API concentration determined via UV-Vis (E2) and HPLC (E3, IBU) as a function of extrusion time of pure excipient in the first cleaning sequence for the investigated APIs.

To quantify the API concentration during the initial sequence of the polymer-based cleaning process, the E2 decrease as a function of pure excipient extrusion time was quantified using a split chemometric model and compared to the off-line HPLC data for the other formulations (Figure 5). The in-line API determination is in good agreement with the off-line measurements. Regardless of the API and initial API concentration, within the first 2 min of the first cleaning sequence (pure excipient), less than 3% of the initial API concentration are present in the extrudate. After 4 min, the API concentration in the extrudate is below the LOD for all three formulations. Hence, a pure excipient extrusion of only 4 min is sufficient to produce an extrudate free of any quantifiable API contamination. Based on this finding, however, it is not clear whether the extrusion equipment does not have any API residue, contributing to subsequent cross-contamination. Therefore, the following sections discuss the CV of the extrusion equipment.

### 3.2. Polymer-Based Cleaning Investigation

In order to determine whether the sampled equipment surfaces were clean or contaminated, the residue acceptance limit L was calculated. In light of the frequently used 10 ppm and 0.001 dose criterion [49,50] discussed in [51,52], the residue acceptance limit calculation was based on toxicological data [53]. It was performed for the swab (L_Swab,E2_ = 0.11 µg·mL^−1^, L_Swab,E3_ = 9.9 µg·mL^−1^ and L_Swab,IBU_ = 9.9 µg·mL^-1^) as well as the rinse tests (L_Rinse,E2_ = 0.015 µg·mL^−1^, L_Rinse,E3_ = 3.1 µg·mL^−1^ and L_Rinse,IBU_ = 15.6 µg·mL^−1^), similarly as in ref. [53]. If the sampled equipment has an API content below L, the surface is considered free from any API residue. For a better understanding of the whole HME process, also the surfaces that did not appear visually clean were analysed [2].

#### 3.2.1. Rinse Test Results

The results of the rinse tests for the elements of each screw zone that were fully surrounded by either pulverulent or molten material (see Section 3.3.1) are summarised in Table 5. The basis of the rinse tests, i.e., the extraction procedures for the APIs in the rinse solvents, revealed slightly different results for each formulation. The extraction procedure for ibuprofen in the water/ethanol mixture was complete, since already after 10 h of extraction 100% of API were recovered from the cleaning polymer. In contrast, after 18 h the extraction procedure for E2 and E3 yielded an API recovery of 89.3 ± 5.5% and 83.5 ± 1.0%, respectively, since rather than dissolving in pure ethanol (which is necessary for extracting the hormones) the cleaning polymer swelled. Therefore, the detected hormone concentrations do not represent 100% of API recovery and, thus, are likely to be higher than the values displayed in Table 5. In general, it can be concluded that as long as the screws are surrounded by powder (zone 1), they are contaminated with API residues. The reason is that neither the pure excipient nor the cleaning polymer melts in the solid conveying zone. Therefore, the screw surface is not fully wetted in the liquid cleaning phase [54]. Additionally, the cleaning polymer does not reveal its highly detergent behaviour as a powder. As soon as the polymer melts due to the shear forces of the kneading blocks (from screw zone 2 onwards), no API can be found on the screws regardless of the formulation investigated. The metallic surfaces of the screw elements are mechanically cleaned by the melt that is mainly transported via a plug and drag flow [55]. Moreover, the surfaces are cleansed due to the detergent effect of the cleaning polymer and its inorganic fillers [23]. Therefore, the surfaces are free of any API residue.

As expected, an increasing API load results in higher API concentrations in the first screw zone. The drastic difference between the ibuprofen and the hormone concentrations in the first rinsed screw zone may be attributed to several factors. First, the different percentage of API recovery from the melt state, as described above, may also play a minor role in the powder state. Secondly, in contrast to Eudragit powder (angle of repose < 10° [56]), EVA flows poorly (angle of repose > 40° [57]). As a result, the EVA-based formulations may tend to form powder chunks that are conveyed towards the kneading blocks more easily than EUD/IBU_10%. Finally, manual irregularities during the screw removal and transfer to the next processing steps may be another cause of the differences in the API contamination in the first screw zone. These hypotheses need to be verified in the future.

#### 3.2.2. Swab Test Results

A summary of all swabbed locations in the extruder after the polymer-based cleaning sequence is provided in Table 6. Since all conveying screw elements have self-cleaning properties [39], only the surfaces in between the screw elements GFF and GFA as well as KB30° and KB60° were investigated (Figure 2b). Despite the use of old screw elements and APIs with a small particle size (X_90,Hormones_ < 10 µm, X_90,IBU_ < 50 µm), negligible API concentrations are detected for all formulations in between adjacent screw elements of one screw. The reason for this is twofold: First, due to the high Van der Waals forces, fine particles (relevant for GFF-GFA) tend to agglomerate and do not fit easily in between the screw elements. Secondly, in the already molten material (relevant for KB30°-KB60°), the viscosity of any polymer-based formulation and the viscous forces are likely too high to allow the material to penetrate through the gap between the adjacent screw elements of one screw [58].

The barrel entrances 1, 6 and 9 are analysed, since they are prone to possible material accumulations due to the geometrical change in the barrel diameter (Figure 1a) and gaps in the thread between the barrel openings and the plug. Regardless of the barrel entrance location, an increasing API content results in higher measured API concentrations (Table 6), with only EUD/IBU_10% indicating critically contaminated barrel entrances. Due to a low API loading of 1 wt.-%, all swab tests on the equipment after extruding EVA/E2_1% are below LOD. Since, similarly to the first screw zone (Section 3.2.1), the pulverulent material in barrel zone 1 can be effectively cleaned neither by the excipient nor the cleaning polymer powder, API contamination at the entrance to the first barrel is likely, particularly at high API loadings. All other barrel openings, e.g., for de-gassing or side-feeding, tend to accumulate the melt and act as dead zones for the extrudate, which are known to be problematic for other pharmaceutical equipment, such as mixers [59] and blenders [31]. In an HME process, the melt can accumulate in the cavities under the closed barrel openings and cannot be actively cleaned either by the excipient or the cleaning polymer, since extensive flow circulations do not occur in these regions. As a consequence, considerable amounts of API residue are found in the dead spots (barrel entrances 6 and 9), especially at high API loadings. In summary, the contaminated dead spots are highly problematic for subsequent extrusions in terms of cross-contamination, since they cannot be cleaned automatically using the polymer-based cleaning sequence.

After the screw removal, the barrel channels of the investigated formulations have both clean and partly contaminated surfaces. In general, however, the same tendency of increasing contamination at higher API loadings, with only EUD/IBU_10% having contaminated barrel channels, can be observed. All contamination in the barrel channels can be introduced by removing the screws that are fully surrounded by the API and the excipient powder in the first screw zone (see Section 3.3.1 and Table 5). During the screw removal, it is highly likely that the API detaches from the screws and sticks to the barrel channel, contaminating it. As a result of the manual screw removal, the exact location of contamination along the barrel channel may be random and depend on the user handling.

### 3.3. Solvent-Based Cleaning Investigation

The polymer-based cleaning was compared to the solvent-based cleaning in the same rinse and swab locations as discussed above. Only EUD/IBU_10% is discussed below, since this formulation has the highest potential for equipment contamination. In addition, it was easier to quantify any changes in API concentration for this formulation due to the higher drug load.

#### 3.3.1. Rinse Test Results

**Figure 6:**
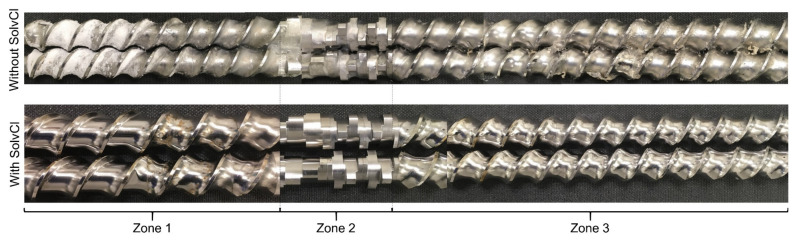
Comparison of the first three screw zones after extruding EUD/IBU_10% via the polymer-based cleaning sequence (without SolvCl) or the solvent-based cleaning sequence (with SolvCl).

After the polymer-based cleaning of EUD/IBU_10%, the screws (Figure 6) appear rather typical for a standard extrusion process with non-degraded polymers [60]. Specifically, the screws contain pulverulent raw materials in the beginning of screw zone 1, which are highly contaminated with the API (Table 7), and API-free melt residue starting from the end of screw zone 1 until the screw tip (not shown in Figure 6). After the solvent-based cleaning sequence, the appearance of the screws is completely different. Neither the powder nor the melt residues can be found on any screw element, and both screws look clean. Since the cleaning polymer fully dissolves in water, any of its residue is effectively washed by water in combination with high shear forces due to the high screw speed of 1200 rpm. As expected, due to the solubilities, large amounts of the remaining IBU were successfully removed using the ethanol/water solution, since the 570 mL of solution that fit into the barrel channel still contained 32.8 µg·mL^−1^ of API. This remaining API content confirms that the API solubilisation step (here via ethanol/water) is essential for a proper cleaning-in-place strategy, and that cleaning the equipment only with water does not suffice. Additionally, this suggests a successful *in-situ* cleaning process of all screw elements, which is confirmed by the HPLC analysis (Table 7). The previously contaminated screw elements in zone 1 are effectively cleaned by the solvent-based cleaning sequence down to API concentrations below LOD.

#### 3.3.2. Swab Test Results

The additional solvent-based cleaning has a significant effect on the areas in between the connecting screw elements of one screw (Table 8). Regardless of the location, the solvent-based cleaning step considerably increases the API concentrations in between the screw elements to above the acceptance limit. This finding is mainly connected to the drastic decrease in the viscosity (η) of the cleaning media of more than six orders of magnitude (η_CleanPoly_ ~ 10^3^ Pa·s at 160 °C, η_Solvent_ < 10^−3^ Pa·s at 60 °C [61,62]). The low viscosity of the solvent facilitates wetting of the screw surfaces [58], allowing the solvent to diffuse into every type of gap. In this regard, a high IBU solubility in the cleaning solvent, which is necessary for an overall effective cleaning, makes every type of gap (such as those in between screw elements) highly vulnerable to API contamination. In addition, since IBU fully dissolves in the ethanol/water solution [45], the path of API transport is not limited by the API’s particle size, but rather by the physical properties of the API-solvent solution and the metallic screw surface with respect to the gap size. Besides a higher viscosity of the solvent [63], an increase in the hydrophobicity of the screw surface or a decrease in the gap size [64] could prevent the solvent from wetting the gaps. In the present case, the screw elements are made of high quality, pharmaceutical-grade stainless steel that generally has good wetting behaviour for water [65] and an even better one for aqueous ethanol solutions [66]. Therefore, the steel type in combination with the solvent employed in the present case promotes wetting in gaps. Additionally, since extensively used screw elements were applied in the present case, it is likely that the sealing of the gap between the adjacent screw elements deteriorated through the addition of scratches with operation time enough to facilitate spreading the API-contaminated solvent into the gaps. Furthermore, the manual tightening of the screw elements onto the shaft might have influenced the gap size between adjacent screw elements of one screw. Although the high centrifugal forces induced by the screw speed of 200 rpm can generally counteract the wetting of surfaces in between the screw elements, high degrees of API contamination are still present on both GFF-GFA and KB30°-KB60° surfaces. Therefore, further studies are required to investigate the effects of screw surfaces and gap sizes in between the adjacent screw elements, e.g., of new screw elements, in terms of API contamination after the solvent-based cleaning sequence. Moreover, in a separate investigation it would be relevant to analyse, whether APIs trapped in between the screw elements can be released into the melt of subsequent extrusions, contributing to cross-contamination.

Another conclusion that can be drawn from these findings is that polymer-based cleaning is required prior to the solvent-based cleaning sequence, particularly if an API solubilisation step is employed. Despite the high API concentrations in the first screw zone (Table 7), most of the API is removed using the pure polymer and the cleaning polymer. Therefore, if the solvent-based cleaning sequence is conducted directly after the API extrusion, every gap in the extrusion equipment near the first screw zone is likely to be contaminated with the API. 

Another interesting finding of the swab tests is the large difference in the API concentrations in the gaps between the screw elements of the first screw zone (GFF-GFA, c_IBU_ = 13.9 µg·mL^−1^) and the second screw zone (KB30°–KB60°, c_IBU_ = 44.4 µg·mL^−1^) after the solvent-based cleaning sequence (Table 8). This can be ascribed to the different material flow in the respective screw elements. Conveying elements with a continuous and unbroken geometry, such as GFA and GFF elements, have a continuous melt transport. In contrast, kneading elements have a discontinuous geometry with typically four or five kneading blocks designed to break up, stretch and fold the flow in order to facilitate sufficient distributive and dispersive mixing and to increase the residence time [22,38,40,67]. This inevitably leads to local accelerated and decelerated flow with lower material transport than that in the conveying elements, which is also valid for the solvent flow during the solvent-based cleaning sequence. Consequently, it is not surprising that the kneading elements have a higher residual API content in between the adjacent screw elements than the conveying elements do.

In contrast to polymer-based cleaning, solvent-based cleaning does not lead to significant API concentrations at the barrel entrance of the main material inlet (barrel 1, Table 8). Similarly to screw zone 1 (Table 7), most of the API is mechanically washed away by the turbulent water flow induced by the spray nozzle and the highly soluble solvent. Yet, the problematic dead spots in barrels 6 and 9 remain contaminated with the API, despite the solvent-based cleaning. If the solvent flow could reach the area of the dead spots, the remaining API would be dissolved, but the solvent would be immediately pressed towards the barrel closure due to the closed exit valve (Figure 1a). As a consequence, the dissolved API would be trapped in the low-tolerance gap between the plug and the barrel similarly to the space in between screw elements. A second possible scenario is that the solvent could not reach the dead spots area due to a low flow circulation. In this case, the dead spots could not be locally cleaned as the opening of barrel 1, which is equipped with the spray nozzle.

Similar to the clean screws (Figure 6), the barrel channels do not contain any critical amount of API after the solvent-based cleaning. Particularly the first barrel zone does not contain any quantifiable API content due to the active washing using the spray nozzle. In contrast to the polymer-based cleaning, no API residue detaches from the clean screws and stick to the barrel channel. Nonetheless, both barrels 5 and 10 have API concentrations that are very close to the residue acceptance limit (L _Swab,IBU_ = 9.9 µg·mL^−1^). This contamination is most likely introduced by removing the neighbouring openings of barrels 6 and 9, i.e., the aforementioned dead spots, and by the swabbing procedure of those barrel entrances, since both steps are performed after the screw removal and before the swabbing procedure of the barrel channels.

#### 3.3.3. Concluding Recommendations

In brief, the novel *in-situ* solvent-based cleaning approach presented in this study has great potential for drastically reducing the manual cleaning effort of pharmaceutical extrusion equipment. To fully eliminate any risk of cross-contaminating subsequently extruded formulations, the presented method has to be further optimised. The process recommendations below only involve minor equipment adaptions, but can potentially lead to major improvements to processing equipment cleaning.

**Figure 7:**
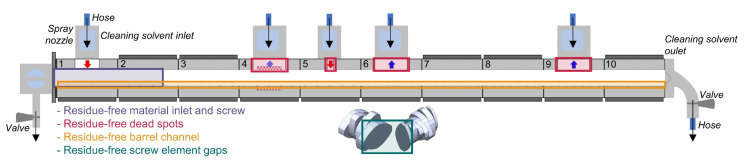
Proposed solvent-based cleaning set-up with a cleaning solvent inlet attached to each barrel opening. The problematic contaminated regions that are cleanable in this recommended set-up are indicated by coloured rectangles.

An additional *in-situ* solvent-based cleaning sequence proved to be very efficient in removing any API residue in the vicinity of the cleaning solvent inlet and the spray nozzle, i.e., the surfaces of the barrel and screw zone 1 (Figure 7). High API concentrations in the problematic dead spots at the barrel openings can be removed by introducing an additional cleaning solvent inlet equipped with a spray nozzle at each barrel opening (Figure 7). If the solvent-based cleaning sequence is performed using many cleaning solvent inlets simultaneously, residue-free dead spots are very likely to be achieved. Although applying numerous cleaning solvent inlets may appear challenging and labour-intensive at first glance, the equipment installation lasts only for a few minutes and is still much faster than manual cleaning. In the proposed cleaning set-up, only the plugs of barrel openings need to be washed manually, while all the other equipment is cleaned *in-situ* in a fully automatic manner.

Another important issue to consider is the necessary number of barrel openings in a pharmaceutical extruder, which can easily be adapted by exchanging selected barrels. Naturally, many barrel openings enable a vast flexibility in terms of multiple feeding and de-gassing, making such an extruder design ideal for research and development purposes. However, a pharmaceutical extruder for the production of drugs should only have the minimum number of barrel openings (in the best case bore holes instead of open cylinders) in order to guarantee an efficient and facile cleanability of the dead spots.

Another possible contamination matter arising from the present study is the disposal of API on the equipment surface during the screw removal. To ensure a residue-free barrel channel, whose surface is most likely contaminated due to an improper screw removal, it is recommended to flush the extruder with the solvent once more after the screw removal.

The last concern associated with the solvent-based cleaning strategy is the API contamination in between non-self-cleaning screw elements of one screw. It can be addressed via various strategies. Firstly, special care should be taken to replace worn-down screw elements, since the gap in between such elements can be significantly larger than it is in new elements, facilitating API accumulations. Secondly, for the tightening of the screw elements onto the shaft, the minimum and maximum torque recommended from the extruder manufacturer should always be considered in order not to introduce gaps between neighbouring screw elements. Thirdly, extruder manufacturers should aim at reducing their tolerances for any type of gap in their equipment that could be in contact with the solvent. This can be achieved by a special polishing step of the elements’ front surfaces or by developing a special gap sealing, which could be applicable to larger screw diameters. Fourthly, in order to reduce wetting of the screw gaps, optional surface treatments to increase the hydrophobicity of screw elements should be considered [65]. Since the centrifugal forces of the screws antagonise the wetting of screw gaps, during the *in-situ* solvent-based cleaning, the screws should never be soaked in the solvent without being rotated. As a final solution to ensure no cross-contamination due to the screw gaps, non-modular screws similar to those used in single-screw extruders could be applied [55].

For further research, it is recommended to limit the maximum screw speed to 800 rpm during the solvent-based cleaning procedure, as a long-term application of higher screw speeds might induce wear, particularly when the lubrication effect of the liquid is not sufficient. In addition, it is recommended to verify potential reductions of API contamination by introducing the API via split-feeding into the polymer melt or as a suspension via a liquid pump. In case a single solvent or solvent mixture that dissolves the API, the polymer and the cleaning polymer is not available, further multi-step solvent-based cleaning approaches that attempt to clean first the thermoplastic residue and in a subsequent step the API, need to be investigated in future research works.

## 4. Conclusions

This study is the first in-depth and systematic investigation of an API-based cleaning process of typical pharmaceutical hot melt extrusion (HME) equipment. In particular, polymer-based and novel *in-situ* solvent-based cleaning sequences were investigated. The application of various characterisation techniques highlighted the cleanability of the equipment from different formulations typical for HME. In this way, innovative insights into the cleaning-in-place strategy for this emerging pharmaceutical production technique were obtained. On the basis of the experiments, the following conclusions were drawn:

- After the first step of polymer-based cleaning, during which only the excipient is extruded, the API loss as a function of extrusion time was determined for the first time by means of inline UV–Vis spectroscopy. Very low API concentrations of down to 0.005 wt.-% were determined for the hormone E2. It was established that regardless of the formulation, already after 4 min of pure excipient extrusion the API concentration in the extrudates was below LOD.
- To obtain equipment without API residue, the polymer-based cleaning sequence additionally featured the extrusion of a cleaning polymer optimised for pharmaceutical HME purposes. This cleaning polymer effectively cleaned the extruder screws of any API as soon as the polymer was molten. However, the first screw and the barrel zone were highly contaminated, particularly in the case of formulations with higher API loadings, since the pulverulent cleaning polymer could not remove the API powder residue. Therefore, special care needs to be taken during the screw removal in order not to contaminate the barrel channel. Additionally, dead spots in the barrels, i.e., the barrel openings for side-feeding or de-gassing, were found to be contaminated due to the lack of flow circulation.
- An attempt to remove an identified API contamination was undertaken by applying a novel solvent-based cleaning sequence in addition to the polymer-based cleaning step. Using special equipment, solvents were introduced into the extruder barrel via spray nozzles, while the extruder operated in a normal manner, i.e., with heated barrels and rotating screws. By using solvents that dissolve both the API and the remaining polymer, the extruder barrels and all zones of the previously highly contaminated screws were effectively cleaned *in-situ* in a fast and facile way. The API accumulations in the dead spots of the barrel openings could not be cleaned properly, which can be counteracted by applying spray nozzles to each barrel opening. However, the solvent-based cleaning approach introduced one issue: Due to its low viscosity, the solvent is more likely to enter gaps in the system, such as in between the adjacent screw elements of one screw. Special care needs to be taken when employing intensively used screw elements, since an API penetration through gaps is likely to be larger than in new systems due to the presence of surface scratches. This issue can be addressed by keeping the screws in rotation during the solvent immersion or using more hydrophobic screw surfaces.

Based on our findings, recommendations were developed on how to effectively and efficiently remove any API residue on pharmaceutical HME equipment, which typically requires extensive manual labour. This study improves the current cleaning strategies for residue-free processing equipment. The proposed cleaning approach can be applied to a wide range of pharmaceutical processing equipment and appears promising in many applications, e.g., in the polymer or food industry or for large extruders that cannot be disassembled easily for cleaning.

## Figures and Tables

**Table 1:** Compositions and designations of the formulations consisting of ethylene-vinyl acetate copolymer with a vinyl acetate content of 28% (EVA), Eudragit RL-PO (EUD), 17β-estradiol (E2), estriol (E3) or ibuprofen (IBU).

Sample Designation	EVA (wt.-%)	EUD (wt.-%)	E2 (wt.-%)	E3 (wt.-%)	IBU (wt.-%)
EVA/E2_1%	99	-	1	-	-
EVA/E3_5%	95	-	-	5	-
EUD/IBU_10%	-	90	-	-	10

**Table 2:** Extrusion and polymer-based cleaning process sequence for EVA/E2_1% and EVA/E3_5% with the settings used for the process time (t_Process_), feeder throughput (ṁ), screw speed (n) and respective extruder barrel temperatures (T_B1-10_) in each step.

Material in the Extruder	t_Process_ (min)	ṁ (kg·h^−1^)	n (rpm)	T_B1_ (°C)	T_B2_ (°C)	T_B3_ (°C)	T_B4_ (°C)	T_B5-10_ (°C)
EVA/E2_1%	30	1.9	300	25	40	80	115	120
EVA/E3_5%							95	95
Polymer-based cleaning sequence								
EVA	10	1.9	300	25	40	80	115	120
	10	3.0	150					
	10	1.5	400					
CleanPoly	5	-	300	25	40	90	160	160
	5	-	150					
	5	-	400					

**Table 3:** Extrusion and the polymer- and solvent-based cleaning process sequence for EUD/IBU_10% with the settings used for the process time (t_Process_), feeder throughput (ṁ), screw speed (n) and respective extruder barrel temperatures (T_B1-10_) in each step.

Material in the Extruder	t_Process_ (min)	ṁ (kg·h^−1^)	n (rpm)	T_B1_ (°C)	T_B2_ (°C)	T_B3_ (°C)	T_B4_ (°C)	T_B5-10_ (°C)
EUD/IBU_10%	30	1.9	300	25	40	80	115	120
Polymer-based cleaning sequence								
EUD	10	1.9	300	25	40	90	160	160
	10	3.0	150					
	10	1.5	400					
CleanPoly	5	-	300	25	40	90	160	160
	5	-	150					
	5	-	400					
Additional solvent-based cleaning sequence								
Water	10	-	1200	25	25	25	25	25
Ethanol/Water (50/50)	20	-	200	60	60	60	60	60
Water	10	-	1200	25	25	25	25	25
None	10	-	0	120	120	120	120	120

**Table 4:** High-performance liquid chromatography (HPLC) methods for the swab and rinse analysis of the investigated active pharmaceutical ingredients (APIs).

Settings	E2	E3	IBU
Stationary phase	Xselect HSS T3 (2.5 µm; 2.1 mm × 100 mm; Waters Corporation, USA) with pre-column		Acquity UPLC BEH C18 (1.7 µm; 2.1 mm × 50 mm; Waters Corporation, USA)
Column temperature (°C)	35	30	30
Mobile phase	50 vol.-% water and 50 vol.-% acetonitrile	67 vol.-% water and 33 vol.-% acetonitrile (gradient)	50 vol.-% chloroacetic acid 0.1M at pH 3.0 and 50 vol.-% acetonitrile
Flow rate (mL·min^−1^)	0.5	0.4	0.5
Injection volume (µl)	4		3
Run time (min)	5	8	4
Detection wavelength (nm)	280		231
Range of linear calibration plot (µg·mL^−1^)	0.1–2.1 (R^2^ = 0.9999)		2.5–300 (R^2^ = 0.9999)
LOD (µg·mL^−1^)	0.013	0.027	0.61
LOQ (µg·mL^−1^)	0.038	0.082	2.04

**Table 5:** API concentration in the solvent after rinsing the screw elements of each screw zone for 18 h for all polymer-based cleaning sequences investigated. Only the underlined values represent the API-contaminated screw elements, since the measured concentrations are above the acceptance limits L (L_Rinse,E2_ = 0.015 µg·mL^−1^, L_Rinse,E3_ = 3.1 µg·mL^−1^ and L_Rinse,IBU_ = 15.6 µg·mL^−1^).

API	Screw Zones					
	1	2	3	4	5	6
c_E2_ (µg·mL^−1^)	0.26	<LOD	<LOD	<LOD	<LOD	<LOD
c_E3_ (µg·mL^−1^)	0.71	<LOD	<LOD	<LOD	<LOD	<LOD
c_IBU_ (µg·mL^−1^)	418.5 *	<LOQ	<LOQ	<LOD	<LOD	<LOD

* above the HPLC calibration range.

**Table 6:** API concentration on the swabbed surfaces in between selected screw elements, on the barrel entrances and in the barrel channels for all polymer-based cleaning sequences investigated. Only the underlined values represent API-contaminated surfaces, since the measured concentrations are above the acceptance limits L (L_Swab,E2_ = 0.11 µg·mL^−1^, L_Swab,E3_ = 9.9 µg·mL^−1^ and L_Swab,IBU_ = 9.9 µg·mL^−1^).

API	In between Screw Elements		Barrel Entrances			Barrel Channel		
	GFF- GFA	KB30°- KB60°	1	6	9	1	5	10
c_E2_ (µg·mL^−1^)	<LOD	<LOD	<LOD	<LOD	<LOD	<LOD	<LOD	<LOD
c_E3_ (µg·mL^−1^)	<LOD	<LOD	0.26	0.25	0.30	0.24	<LOD	0.31
c_IBU_ (µg·mL^−1^)	8.9	<LOD	20.3	38.6	27.8	4.8	20.4	26.4

**Table 7:** Comparison of the rinse results for each screw zone between the polymer- and solvent-based cleaning sequences for the formulation EUD/IBU_10%. Only the underlined values represent the API-contaminated screw elements, since the measured concentrations are above the acceptance limit L (L_Rinse,IBU_ = 15.6 µg·mL^−1^).

API	Cleaning	Screw Zones					
		1	2	3	4	5	6
c_IBU_ (µg·mL^−1^)	Without SolvCl	418.5 *	<LOQ	<LOQ	<LOD	<LOD	<LOD
	With SolvCl	<LOD	<LOD	<LOD	<LOD	<LOD	<LOD

* above the HPLC calibration range.

**Table 8:** Comparison of the swab results between the polymer- and solvent-based cleaning sequences for EUD/IBU_10%. Only the underlined values represent the API-contaminated surfaces, since the measured concentrations are above the acceptance limit L (L_Swab,IBU_ = 9.9 µg·mL^−1^).

API	Cleaning	In between Screw Elements		Barrel Entrances			Barrel Channel		
		GFF- GFA	KB30°- KB60°	1	6	9	1	5	10
c_IBU_ (µg·mL^−1^)	Without SolvCl	8.9	<LOD	20.3	38.6	27.8	4.8	20.4	26.4
	With SolvCl	13.9	44.4	5.3	49.2	14.4	<LOD	9.2	9.6
